# Draft genome announcement of *Bacillus velezensis* TSB6.1 isolated as a culturable endosymbiont of a nitrogen-fixing endophytic yeast *Rhodotorula mucilaginosa* JGTA-S1

**DOI:** 10.1128/mra.01202-23

**Published:** 2024-06-12

**Authors:** Mayurakshi Nag, Anindita Seal

**Affiliations:** 1 Department of Biotechnology and Dr B. C. Guha Centre for Genetic Engineering and Biotechnology, University of Calcutta, Kolkata, West Bengal, India; University of Maryland School of Medicine, Baltimore, Maryland, USA

**Keywords:** *Rhodotorula mucilaginosa *JGTA-S1, endosymbiont, *Bacillus velezensis*

## Abstract

We here report the genome of *Bacillus velezensis* TSB6.1 isolated as a culturable endosymbiont of an endophytic yeast *Rhodotorula mucilaginosa* JGTA-S1. TSB6.1 has a genome size of approximately 4.50 Mb, with 4,597 genes, 45.54% GC content, 3 rRNAs, and 73 tRNAs.

## ANNOUNCEMENT

*Bacillus velezensis* TSB6.1, is a rod-shaped, gram-positive, biofilm-forming endosymbiotic bacterium isolated from a basidiomycetous, nitrogen-fixing yeast *Rhodotorula mucilaginosa* JGTA-S1 that improves nitrogen nutrition in rice plants (1). JGTA-S1 was isolated as an endophyte of the wetland plant *Typha angustifolia*, which grows in a uranium mine-associated wetland situated in Jaduguda, India.

To isolate culturable endosymbionts of JGTA-S1, it was cultured in tryptic soy broth (TSB) for 48 h at 28°C. The cell pellet was washed thrice with autoclaved ultrapure water. The cells were stained with lactophenol cotton blue and examined by microscopy before they were ruptured using sterile glass beads in PBS buffer (pH 7.4). Cell extract was plated on TSB agar plates. We selected a colony with diffused, whitish colony morphology to obtain a pure culture. Endosymbionts in unicellular organisms like yeasts are rarely described (2). To the best of our knowledge, this is the first report of an endosymbiotic *Bacillus velezensis* genome of the unicellular fungus *R. mucilaginosa* JGTA-S1.

TSB6.1 was grown in TSB for 24 h at 28°C. To isolate bacterial genomic DNA, lysozyme treatment followed by SDS method was performed. CTAB-NaCl solution was added to get better DNA yield (https://jgi.doe.gov/wp-content/uploads/2014/02/JGI-Bacterial-DNA-isolation-CTAB-Protocol-2012.pdf). A paired-end library (2 × 150 bp) was prepared from the genomic DNA using the NEBNext Ultra DNA Library Preparation Kit and used for sequencing on the Illumina Hiseq2500 platform.

**Fig 1:**
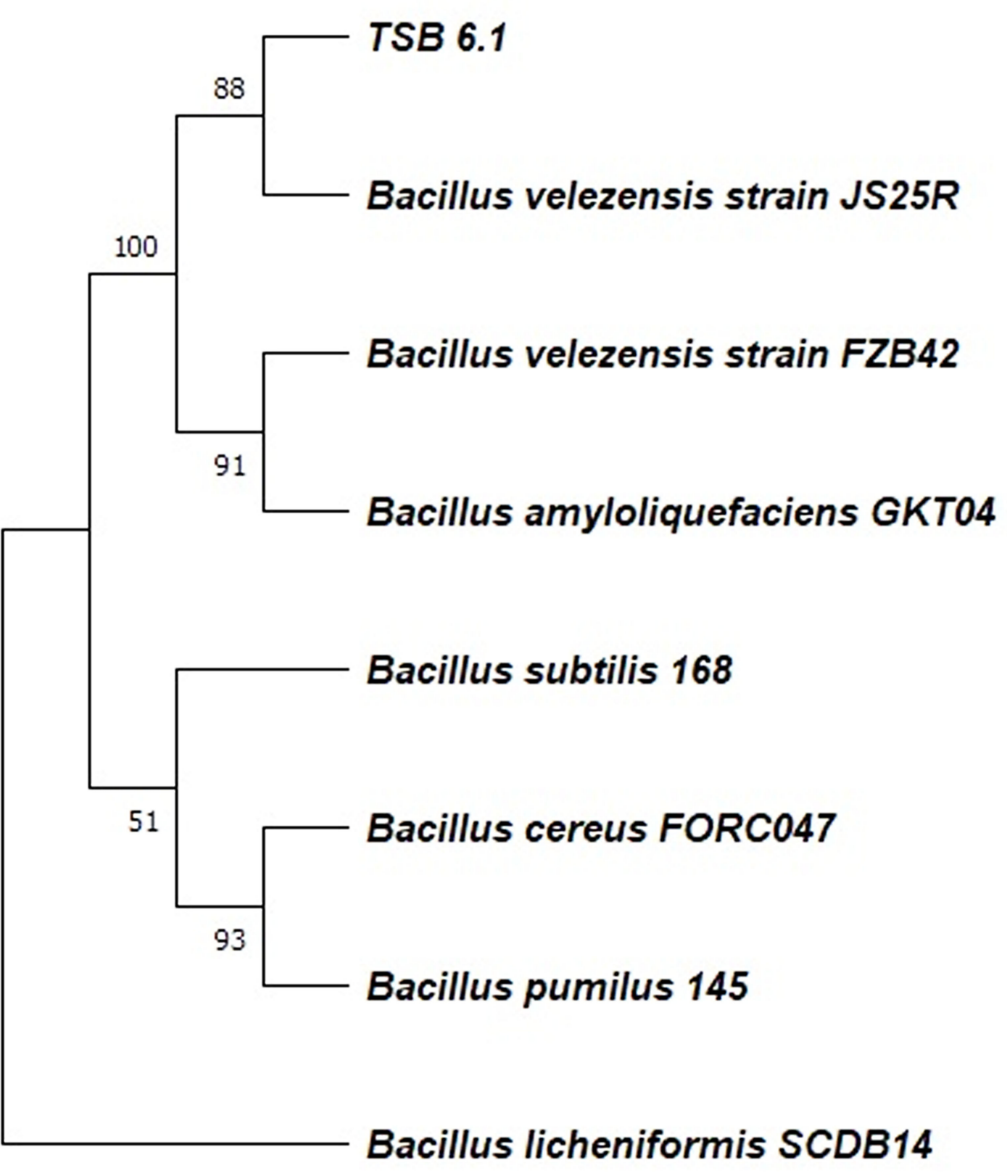
Phylogenetic relationship of TSB6.1 with the other *Bacillus* sp. Ftsz genes from the selected strains were aligned using MUSCLE, and then phylogenetic tree was generated using neighbor-joining method with 5,000 bootstrap replicates in MEGA11. Bootstrap values are represented at the nodes of the branches.

The FASTQ file was pre-processed using AdapterRemoval v2.3.1 (3) to filter out adapter sequences, and any paired-end reads with an average quality score of less than 30 were discarded. *De novo* assembly was performed using Unicycler v0.4.8 (4). Genome completeness and contamination were checked using CheckM v1.0.18 (5) (Table 1). Average nucleotide identity (ANI) score of 98.29% was found when TSB6.1 genome was compared with *B. velezensis* FZB42 using the ANI calculator with its default parameters (6). For most *Bacillus* sp., type strains were chosen for phylogenetic tree construction. A conserved cell division gene FtsZ genes from the selected strains were aligned using MUSCLE and subjected to tree construction using the neighbor-joining method in MEGA11 (7) (Fig. 1) (with 5,000 bootstrap replicates). TSB6.1 genome size is approximately 4.50 Mb, 4,597 genes, 23 pseudogenes, and 45.54% GC content. The genome was annotated using PGAP (8) (JAOXLR000000000).

**TABLE 1:** Summary of the draft whole-genome sequence of *Bacillus velezensis* TSB 6.1, a culturable endosymbiont of *Rhodotorula mucilaginosa* JGTA-S1

	*Bacillus velezensis* TSB 6.1
Sequencing method	Illumina HiSeq 2500
Illumina reads	26,149,592
No. of contigs ≥200 bp	99
No. of contigs ≥50,000 bp	20
Largest contig	616,665
Total length (bp)	4,501,860
*N*50	319,394
GC%	45.54
Completeness	99.81
Contamination	0.89
Sequencing coverage	56×
Complete and single-copy BUSCO	124
Total BUSCO searched	124
Biosample accession no.	SAMN31310229

Although TSB6.1 is an endosymbiont of a nitrogen-fixing yeast, no *nif* cluster within its genome was found. The genome encodes secondary metabolites, such as bacilysin, difficidin, and bacillaene, which may protect plants by inducing systematic acquired resistance. This genome supports the biofilm-forming ability of TSB6.1.

## Data Availability

The assembled genome has been deposited to NCBI with accession number GCF_025792155.1. The raw reads were submitted into SRA with run number SRR21928706.
